# USP49 negatively regulates cellular antiviral responses via deconjugating K63-linked ubiquitination of MITA

**DOI:** 10.1371/journal.ppat.1007680

**Published:** 2019-04-03

**Authors:** Liya Ye, Qiang Zhang, Tianzi Liuyu, Zhigao Xu, Meng-Xin Zhang, Min-Hua Luo, Wen-Bo Zeng, Qiyun Zhu, Dandan Lin, Bo Zhong

**Affiliations:** 1 Department of Gastrointestinal Surgery, Medical Research Institute, Zhongnan Hospital of Wuhan University, Wuhan, China; 2 College of Life Sciences, Wuhan University, Wuhan, China; 3 Department of Pathology, Center for Pathology and Molecular Diagnostics, Zhongnan Hospital of Wuhan University, Wuhan, China; 4 State Key Laboratory of Virology, CAS Center for Excellence in Brain Science and Intelligence Technology (CEBSIT), Wuhan Institute of Virology, Chinese Academy of Sciences, Wuhan, China; 5 State Key Laboratory of Veterinary Etiological Biology, Lanzhou Veterinary Research Institute, Chinese Academy of Agricultural Sciences, Lanzhou, China; 6 Cancer Center, Renmin Hospital of Wuhan University, Wuhan, China; University of Southern California, UNITED STATES

## Abstract

Mediator of IRF3 activation (MITA, also known as STING and ERIS) is an essential adaptor protein for cytoplasmic DNA-triggered signaling and involved in innate immune responses, autoimmunity and tumorigenesis. The activity of MITA is critically regulated by ubiquitination and deubiquitination. Here, we report that USP49 interacts with and deubiquitinates MITA after HSV-1 infection, thereby turning down cellular antiviral responses. Knockdown or knockout of USP49 potentiated HSV-1-, cytoplasmic DNA- or cGAMP-induced production of type I interferons (IFNs) and proinflammatory cytokines and impairs HSV-1 replication. Consistently, *Usp49*^-/-^ mice exhibit resistance to lethal HSV-1 infection and attenuated HSV-1 replication compared to *Usp49*^+/+^ mice. Mechanistically, USP49 removes K63-linked ubiquitin chains from MITA after HSV-1 infection which inhibits the aggregation of MITA and the subsequent recruitment of TBK1 to the signaling complex. These findings suggest a critical role of USP49 in terminating innate antiviral responses and provide insights into the complex regulatory mechanisms of MITA activation.

## Author summary

Mediator of IRF3 activation (MITA) is an essential adaptor protein in mediating innate immune responses to DNA viruses. The activation of MITA requires K63-linked ubiquitination and oligomerization which promotes the recruitment of downstream kinases and phosphorylation of transcription factors to induce expression of downstream genes. In our study, we have discovered that the deubiquitinating enzyme USP49 interacts with and removes K63-linked polyubiquitin chains from MITA to downregulate antiviral signaling. USP49 deficiency results in enhanced production of antiviral cytokines and attenuated virus replication after HSV-1 infection in cells and *in vivo*. Our study thus reveals a negative feedback regulation of MITA activity and cellular antiviral responses.

## Introduction

The innate immune system is the first line of defense against invading pathogens and innate immune response to microbial species is initiated by the recognition of pathogen-associated molecular patterns (PAMPs) by germline-encoded pattern-recognition receptors (PRRs)[1]. Viral nucleic acid including RNA, DNA and RNA-DNA hybrid constitutes classical PMAPs that are detected by Toll-like receptors (TLRs), retinoic acid–inducible gene I protein (RIG-I)-like receptors (RLRs) and a variety of cytoplasmic DNA sensors [2–5]. Upon binding to the PAMPs, these PRRs recruit adaptor proteins or catalyze second messenger molecules for adaptor proteins activation, trigger a series of signaling cascades and eventually induce expression of an array of downstream genes such as type I interferons (IFNs) and proinflammatory cytokines to elicit antiviral immune responses.

Mediator of IRF3 activation (MITA, also known as STING and ERIS) is an adaptor protein essential for cytoplasmic DNA-triggered signaling and host defense against HSV-1 and *Listeria monocytogenes* [6–10]. Structural and biochemical studies demonstrate that MITA is activated by binding to cyclic dinucleotides such as cyclic di-GMP which is generated by invading bacteria and cyclic GMP-AMP (cGAMP) which is generated by cGAMP synthase (cGAS) upon binding to cytoplasmic DNA either from invading DNA viruses and retroviruses or from self-damaged genomic or mitochondrial DNA [11–18]. In addition, several studies have shown that an array of cytoplasmic DNA sensors such as DAI, IFI16 and DDX41 directly recruit and activate MITA in a ligand and/or cell-type specific manner and the mechanisms are less clear [19–21]. Activated MITA undergoes oligomerization and traffics to the ER-Golgi intermediate compartment (ERGIC) to activate the transcription factors IRF3 and NF-κB, in which iRhom2 plays an essential role [22, 23]. Activated IRF3 and NF-κB enter nucleus to initiate transcription of a large number of downstream genes.

In addition to mediating immune responses against viruses or bacteria, MITA is also implicated in autoimmunity and tumor immunity. Various gain-of-function mutants and noncoding SNPs of MITA are found in patients with MITA-associated vasculopathy with onset in infancy (SAVI) and systemic inflammatory or autoimmune diseases characterized by elevated expression of type I IFNs, IFN-stimulated genes (ISGs) and proinflammatory cytokines [24–27]. Ablation of MITA in *Trex1*^-/-^ mice or *Rnaseh2* loss-of-function mutation knock-in mice significantly reduces systemic inflammation and lethality [28–30]. In contrast, inflammatory responses are significantly elevated by deficiency of MITA in the model of MRL. Fas^lpr^ lupus and 2,6,10,14-tetramethylpentadecane (TMPD)-mediated peritonitis as a result of diminished MITA-mediated expression of suppressive molecules [31], indicating that proper activity and availability of MITA are essential for the balance of protective immunity and excessive autoimmunity. In addition, it has been observed that MITA is activated by tumor DNAs in APCs to promote type I IFN induction, tumor antigen cross presentation and CD8^+^ T cell activation and therapeutic activation of MITA results in plausible tumor regression [32]. However, agonist-mediated abnormal activation of MITA may also contribute to induction of ISGs in tumor cells previously linked with chemoresistance [33–35]. Thus, the activity of MITA should be strictly regulated under physiological conditions and normalization of MITA activity may contribute to improved outcome of anti-tumor immunity or autoimmunity.

Various posttranslational modifications have been reported to modulate the activity of MITA. TBK1 phosphorylates the pLxLS motif of MITA which leads to the recruitment and subsequent phosphorylation of IRF3, whereas ULK1 phosphorylates MITA to promote its degradation [36, 37]. TRIM38 and SENP2 mediate sumoylation and desumoylation of MITA which stabilize MITA and promote the chaperone-mediated autophagy (CMA) pathway-dependent degradation of MITA at the early and late phase of HSV-1 infection, respectively [38]. The E3 ubiquitin ligases including RNF5, TRIM30α and TRIM29 catalyze K48-linked ubiquitination and degradation of MITA [39–42] whereas AMFR promotes K27-linked ubiquitination and activation of MITA [43], which are reversed and controlled by USP18 or USP21 and USP13, respectively [44–46]. Alternatively, MITA undergoes K63-linked ubiquitination by TRIM56, TRIM32 or MUL1 for activation [47–49]. Whether and how such a modification of MITA is regulated remain to be investigated.

Deubiquitinating enzymes counteract with ubiquitination by cleaving poly- or mono-ubiquitin from target proteins and play essential roles in various physiological processes [50]. In an unbiased screening of MITA-interacting DUBs, we have found that USP49 interacts with MITA [45]. USP49 has been reported to deubiquitinate H2B, FKBP51, p53 and DUSP1 and function in pre-mRNA splicing, tumorigenesis and ischemia-reperfusion-induced cell viability [51–54]. In this study, we found that USP49 deconjugates K63-linked polyubiquitin chains from MITA and thereby inhibits the oligomerization and subsequent recruitment of TBK1 after HSV-1 infection. Consistently, USP49 deficiency potentiates HSV-1- or cytoplasmic DNA-triggered signaling and impairs replication of HSV-1 *in vitro* and *in vivo*. These findings uncover a complex regulatory mechanism of MITA by USP49 to prevent excessive immune responses and uncontrolled inflammation.

## Results

### USP49 interacts with MITA and inhibits MITA-mediated signaling

**Fig 1:**
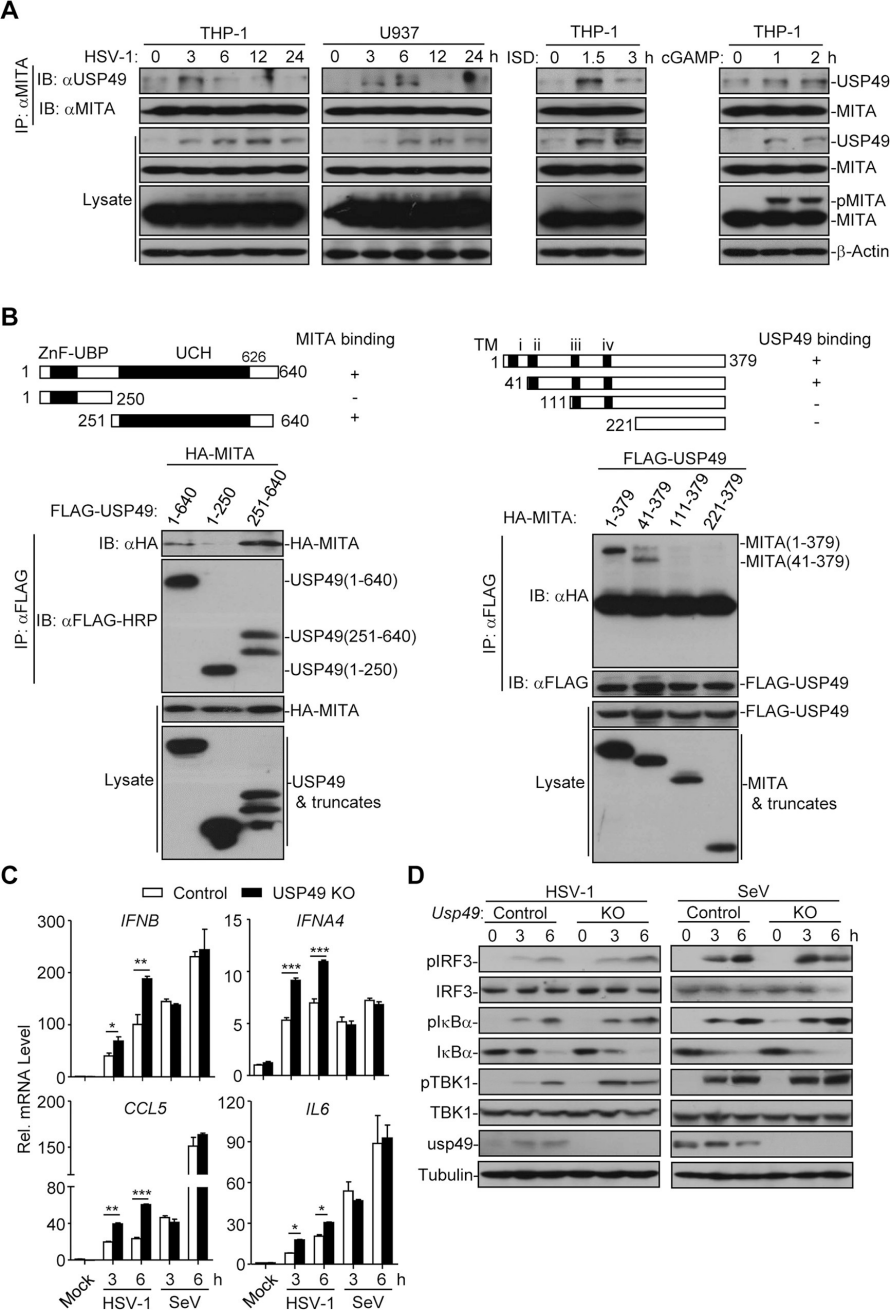
USP49 interacts with MITA and inhibits MITA-mediated signaling. (A) Immunoprecipitation (with anti-MITA) and immunoblot analysis (with anti-MITA or anti-USP49) of THP-1 or U937 cells infected with HSV-1, or transfected with ISD (10 μg) or cGAMP (4 μg) for the indicated time points. (B) Immunoprecipitation (with anti-Flag) and immunoblot analysis (with anti-FLAG or anti-HA) of HEK293 cells transfected with plasmids encoding HA-MITA and FLAG-tagged USP49 or USP49 truncates for 24 h or transfected with plasmids encoding FLAG-USP49 and HA-MITA or MITA truncates for 24 h. (C) qRT-PCR analysis of *IFNB*, *IFNA4*, *CCL5* and *IL6* in control and USP49 KO THP-1 cells infected with HSV-1 or SeV for 0–6 h. (D) Immunoblot analysis of phosphorylated and total IRF3, IκBα, TBK1 and Tubulin in control and USP49 KO THP-1 cells infected with HSV-1 or SeV for 0–6 h. *P < 0.05; **P < 0.01; ***P <0.001 (analysis of two-way ANOVA followed by Bonferroni post-test). Data are representative of three independent experiments (mean ± S.D. in C).

MITA is a critical adaptor protein involved in innate immune signaling, autoimmunity and tumor immunity and the ubiquitination of MITA critically regulates its activation and stabilization [55]. We thus speculated that DUBs-mediated deubiquitination of MITA was as equally important. To test our hypothesis, we conducted an unbiased screening of MITA-interacting DUBs by cotransfection and immunoprecipitation assays and found that USP49 interacted with MITA [45]. Endogenous immunoprecipitation analysis further confirmed that there was an association between USP49 and MITA in human monocytic THP-1 and U937 cells and human foreskin fibroblast (HFF) after HSV-1 infection or transfection of interferon-stimulating DNA (ISD) or cGAMP (*Figs 1A and S1A*). Domain mapping analysis suggested that the UCH domain of USP49 and the second transmembrane domain (aa41-110) of MITA are responsible for their association (*Fig 1B).* Results from reporter assays suggested that overexpression of USP49 inhibited MITA- or cGAS and MITA- but not TBK1- or IRF3-mediated activation of ISRE, whereas knockdown of USP49 potentiated MITA- but not IRF3-mediated activation of ISRE (*S1B and S1C Fig*), indicating that USP49 might function at the level of MITA to regulate innate antiviral signaling.

To further confirm the regulatory role of USP49 in cellular antiviral signaling, we generated USP49 KO THP-1 cells by CRISPR/Cas9-mediated gene editing. As shown in Fig 1C and 1D, knockout of USP49 in THP-1 cells significantly potentiated the expression of *IFNB*, *IFNA4*, *CCL5* and *IL6* and the phosphorylation of IRF3, IκBα and TBK1 after HSV-1 infection. In contrast, SeV-induced *IFNB*, *IFNA4*, *CCL5* and *IL6* and phosphorylation of IRF3, IκBα and TBK1 were not affected by knockout of USP49 in THP-1 cells (*Fig 1C and 1D*). These data together suggest that USP49 interacts with MITA and inhibits MITA-mediated signaling.

### Knockout of USP49 potentiates HSV-1-triggered signaling

To further investigate the role of USP49 in antiviral signaling *in vivo*, we generated USP49-deficient mice by CRISPR/Cas9-mediated genome editing (*S2A Fig*). Results from sequencing showed that there was a deletion of 182 bp of the third exon of *Usp49* gene, which caused a reading frame shift and led to an early translational termination of USP49 (*S2B Fig*). The *Usp49*^-/-^ mice bred normally with the Mendelian inheritance ratio and did not show any developmental defect until 16-week old compared to the wild-type littermates (*S2B Fig*). The numbers and percentages of various immune cells in thymus, spleen or peripheral lymph nodes were comparable between the *Usp49*^+/+^ and *Usp49*^-/-^ mice (*S2C–S2E Fig).* The differentiation of *Usp49*^-/-^ bone marrow cells into BMDCs and BMDMs was similar to the wild-type counterparts in the presence of GM-CSF and M-CSF, respectively (*S2F Fig*), indicating that USP49 is dispensable for development and homoeostasis of immune cells.

**Fig 2:**
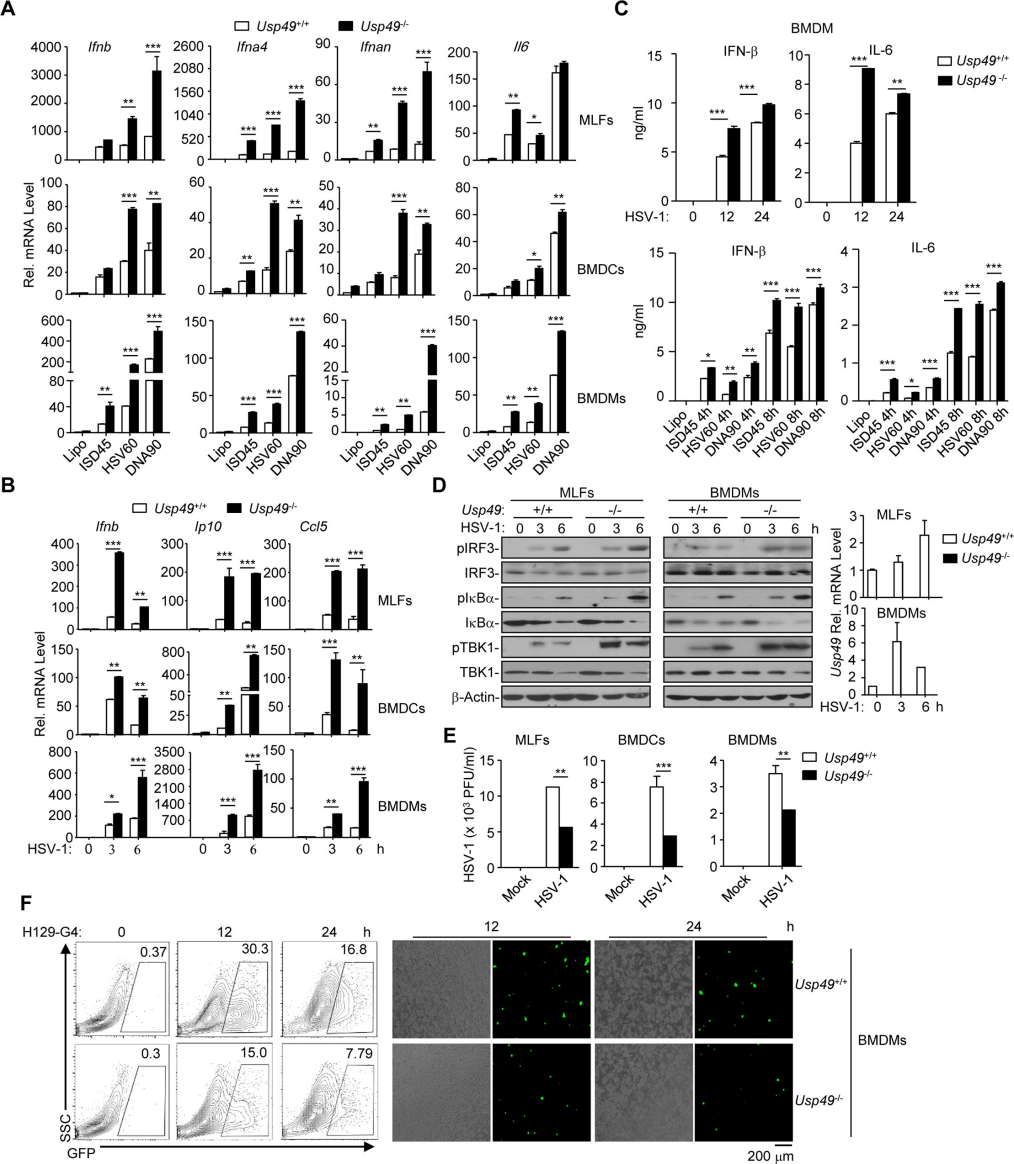
Knockout of USP49 potentiates HSV-1-triggered signaling. (A) qRT-PCR analysis of *Ifnb*, *Ifna4*, *lfnan* and *Il6* mRNA in *Usp49*^+/+^ and *Usp49*^-/-^ MLFs (upper), BMDCs (middle) and BMDMs (lower) left mock transfected (Lipo) or transfected with ISD45, HSV60, DNA90 for 0–6 h. (B) qRT-PCR analysis of *Ifnb*, *IP10*,and *Ccl5* mRNA in *Usp49*^+/+^ and *Usp49*^-/-^ MLFs (upper), BMDCs (middle) and BMDMs (lower) infected with HSV-1 for 0–6 h. (C) ELISA analysis of IFN-β and IL-6 in the supernatants of *Usp49*^+/+^ and *Usp49*^-/-^ BMDMs infected for 0–24 hours or mock transfected (Lipo) or transfected with ISD45, HSV60, DNA90 for 4–8 h. (D) Immunoblot analysis of phosphorylation of IRF3, IκBα, TBK1, total IRF3, IκBα, TBK1 and β-Actin in *Usp49*^+/+^ and *Usp49*^-/-^ MLFs (left) and BMDCs (middle) infected with HSV-1 for 0–6 hours. qRT-PCR analysis of *Usp49* mRNA in *Usp49*^+/+^ and *Usp49*^-/-^ MLFs and BMDCs infected with HSV-1 for 0–6 h (right). (E) Plaque assay of HSV-1 replication in the supernatants of *Usp49*^+/+^ and *Usp49*^-/-^ MLFs, BMDCs and BMDMs infected with HSV-1(MOI of 0.4) for 1 h followed by twice PBS wash and cultured in full medium for 60 h. (F) Flow cytometry analysis (left), microscopy imaging (right) of *Usp49*^+/+^ and *Usp49*^-/-^ BMDMs infected with H129-G4 for 0–24 h. *P < 0.05; **P < 0.01; ***P <0.001 (analysis of two-way ANOVA followed by Bonferroni post-test). Data are representative of three independent experiments (mean ± S.D. in A-E).

We next examined virus-triggered induction of downstream genes in *Usp49*^+/+^ and *Usp49*^-/-^ cells. Interestingly, HSV-1-, cytoplasmic DNA- or cGAMP- but not SeV-induced expression of *Ifnb*, *Ifna4*, *Ifnan* or *Il6* and the production of IFN-β and IL-6 were enhanced in *Usp49*^-/-^ BMDCs, BMDMs and MLFs compared to the *Usp49*^+/+^ counterparts (*Figs 2A–2C and S3 and S4A*). Consistent with these observations, HSV-1- but not SeV-induced phosphorylation of IRF3, IκBα and TBK1 was potentiated in *Usp49*^-/-^ BMDMs and MLFs compared to the *Usp49*^+/+^ counterparts (*Figs 2D and S4B*). Moreover, the replication of HSV-1 or H129-G4 (a GFP-tagged HSV-1) [56] was suppressed in *Usp49*^-/-^ BMDCs, BMDMs or MLFs compared to the *Usp49*^+/+^ counterparts as monitored by the HSV-1 titers in the supernatants and the GFP percentages or intensities (*Fig 2E and 2F*). These data together illustrate that USP49 negatively regulates DNA virus-triggered signaling in various primary mouse cells.

### *Usp49*^-/-^ mice exhibit increased resistance to lethal HSV-1 infection

**Fig 3:**
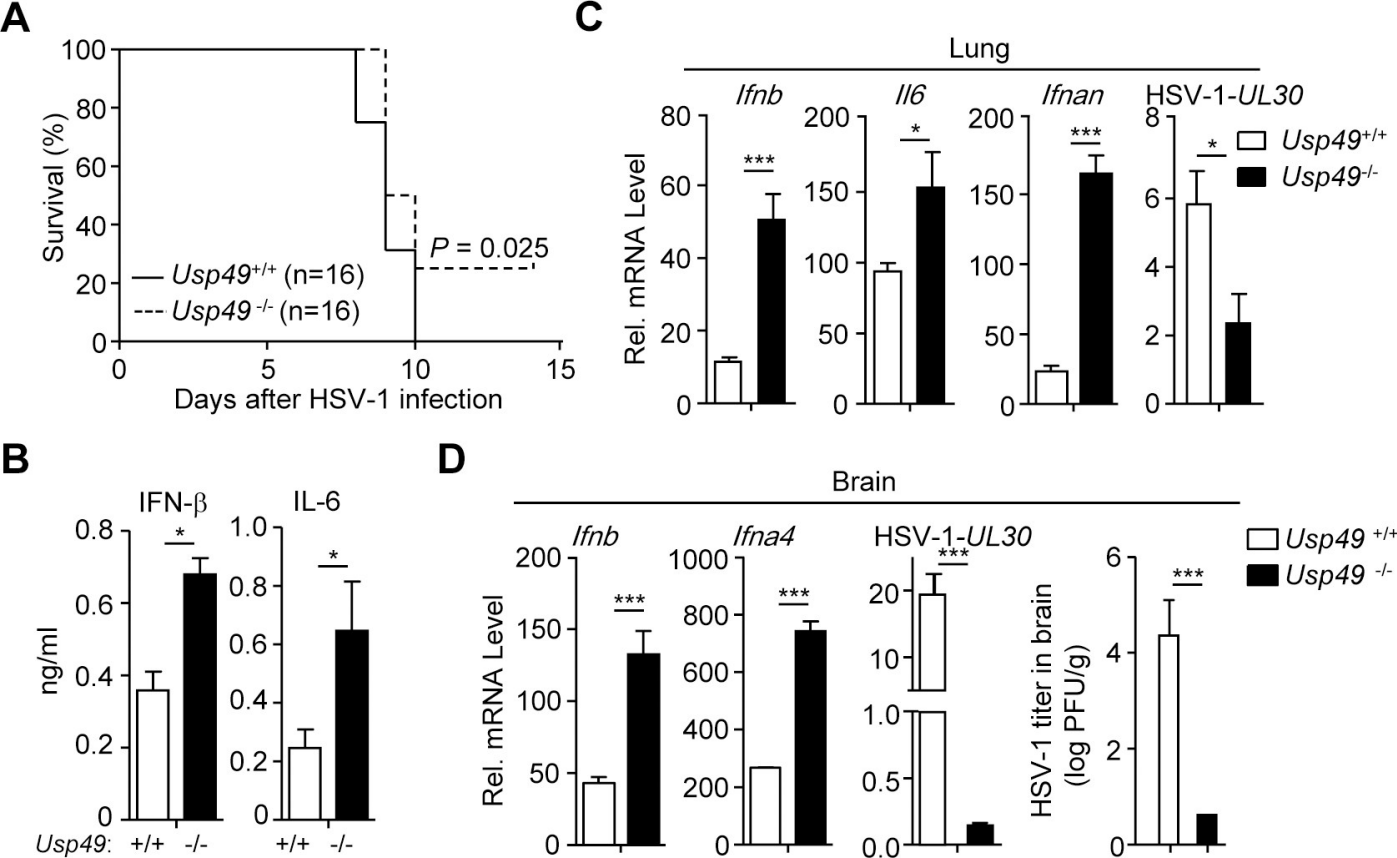
*Usp49*^-/-^ mice exhibit resistance to lethal HSV-1 infection. (A) Survival (Kaplan-Meier curve) of *Usp49*^+/+^ (n = 16) and *Usp49*^-/-^ (n = 16) intravenously injected with HSV-1 (2.5×10^6^ PFU per mouse) monitored survival for 14 days. (B) ELISA analysis of IFN-β and IL-6 in the serum of *Usp49*^+/+^ and *Usp49*^-/-^ mice(n = 3) intravenously injected with HSV-1 (2.5×10^6^ PFU per mouse) for 12 hours. (C) qRT-PCR analysis of *Ifnb*, *Il6*, *Ifnan* and HSV-1-*UL30* mRNA in the lungs (n = 3) from *Usp49*^+/+^ and *Usp49*^-/-^ mice intravenously injected with HSV-1 (2.5×10^6^ PFU per mouse) for 24 hours. (D) qRT-PCR analysis of *Ifnb*, *Ifna4*, HSV-1-*UL30* mRNA and plaque assay of the brains (n = 3) from *Usp49*^+/+^ and *Usp49*^-/-^ mice intranasally injected with HSV-1 (2.5×10^6^ PFU per mouse) for 4 days. *P < 0.05; **P < 0.01; ***P <0.001 (two-tailed t-test). Data are representative of three independent experiments (mean ± S.D. in B-D).

To investigate the function of USP49 in host defense against virus infection *in vivo*, we monitored survival of *Usp49*^+/+^ and *Usp49*^-/-^ mice after intravenous (i.v.) injection of HSV-1. As shown in Fig 3A, *Usp49*^-/-^ mice exhibited a later onset of death and a higher survival rate compared to the wild-type littermates. All the wild-type mice exhibited severe lethargy and died within 10 days. In contrast, 25% (4 out of 16) *Usp49*^-/-^ mice survived after injection (*Fig 3A*). Consistently, the concentrations of IFN-β and IL-6 in the sera of *Usp49*^-/-^ mice were significantly increased compared to those in the *Usp49*^+/+^ littermates 12 hours after HSV-1 infection (*Fig 3B*). Furthermore, we observed that the expression of *Ifnb*, *Il6*, and *Ifnan* was potentiated and the expression of HSV-1-*UL30* gene was inhibited in the lungs from *Usp49*^-/-^ mice compared to *Usp49*^+/+^ mice at 24 hours after HSV-1 infection (*Fig 3C*). In addition, we analyzed the expression of cytokines and viral titers in the brains 4 days after HSV-1 infection. The results showed that the expression of *Ifnb* and *Ifna4* was increased, whereas the expression of HSV-1-*UL30* gene and the replication of HSV-1 was suppressed in the brains from *Usp49*^-/-^ mice compared to those from *Usp49*^+/+^ mice (*Fig 3D*). Collectively, these data demonstrate that loss of USP49 protects mice from HSV-1 infection by promoting the induction of type I IFNs and proinflammatory cytokines.

### The DUB activity of USP49 is required for inhibition of HSV-1-triggered signaling

**Fig 4:**
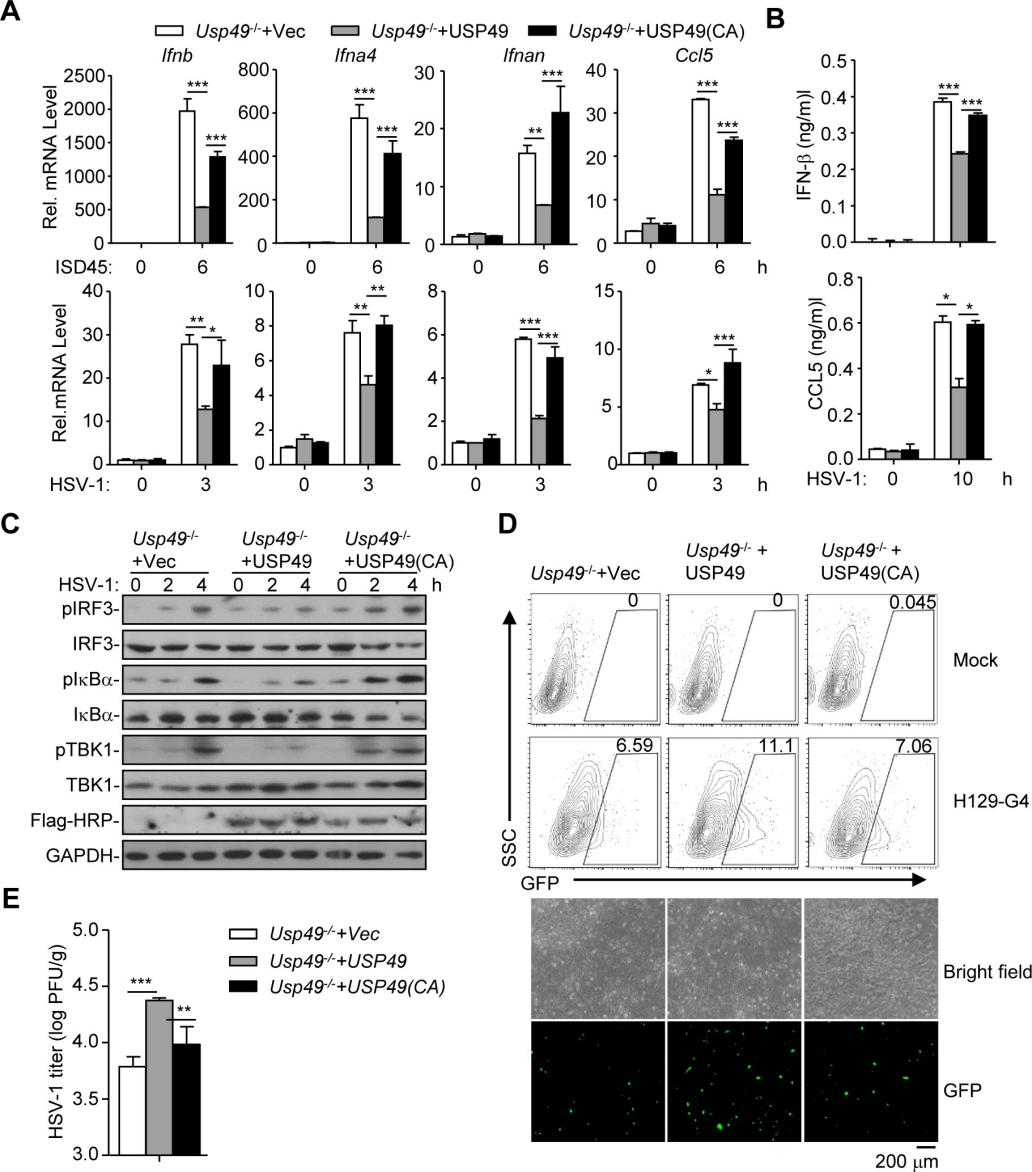
The DUB activity of USP49 is required for inhibition of HSV-1-triggered signaling. (A, B) qRT-PCR analysis of *Ifnb*, *Ifna4*, *Ifnan* and *Ccl5* mRNA (A), and ELISA analysis of IFN-β, Ccl5 (B) in *Usp49*^-/-^ mice MLFs reconstituted with empty vector (*Usp49*^-/-^ +vec), *Usp49* (*Usp49*^-/-^ + Usp49) or *Usp49CA* (*Usp49*^-/-^ + Usp49(CA)) infected with ISD45 for 0–6 hours (A), HSV-1 for 0–3 hours (A) or 0–10 hours (B). (C, D) Immunoblot (C), flow cytometry(D, upper graphs), microscopy imaging (D, lower graphs) and plaque assay (E) of *Usp49*^-/-^ mice MLFs reconstituted with empty vector (*Usp49*^-/-^ +vec), *Usp49* (*Usp49*^-/-^ + *Usp49*) or *Usp49CA* (*Usp49*^-/-^ + *Usp49(CA)*) infected with HSV-1 for 0–4 hours (C), 12 hours (D) or 1 hour (E). *P < 0.05; **P < 0.01; ***P <0.001 (analysis of two-way ANOVA followed by Bonferroni post-test). Data are representative of two (C) or three (A, B, D, E) independent experiments (mean ± S.D. in A, B, E).

We next examined whether the enzyme activity of USP49 was required for the suppression of antiviral signaling. The empty vector, USP49 or its enzymatic inactive mutant USP49 (C262A) was reconstituted into *Usp49*^-/-^ cells followed by HSV-1 infection. Results from qRT-PCR and ELISA analysis showed that HSV-1-induced expression of *Ifnb*, *Ifna4*, *Il6* or *Ccl5* and the production of IFN-β and CCL5 were significantly inhibited in *Usp49*^-/-^ MLFs reconstituted with USP49 but not in those reconstituted with USP49 (C262A) (*Fig 4A and 4B*). Moreover, we found that HSV-1-induced phosphorylation of IRF3, IκBα and TBK1 was impaired by reconstitution of USP49 but not USP49 (C262A) into *Usp49*^-/-^ MLFs (*Fig 4C*). Consistently, replication of HSV-1 was potentiated in *Usp49*^-/-^ MLFs reconstituted with USP49 but not in those reconstituted with USP49 (C262A) as monitored by the GFP signals or HSV-1 titers in the supernatants (*Fig 4D and 4E*). These data suggest that the DUB activity of USP49 is required for inhibition of HSV-1 triggered signaling.

### USP49 deconjugates K63-linked ubiquitination and aggregation of MITA

**Fig 5:**
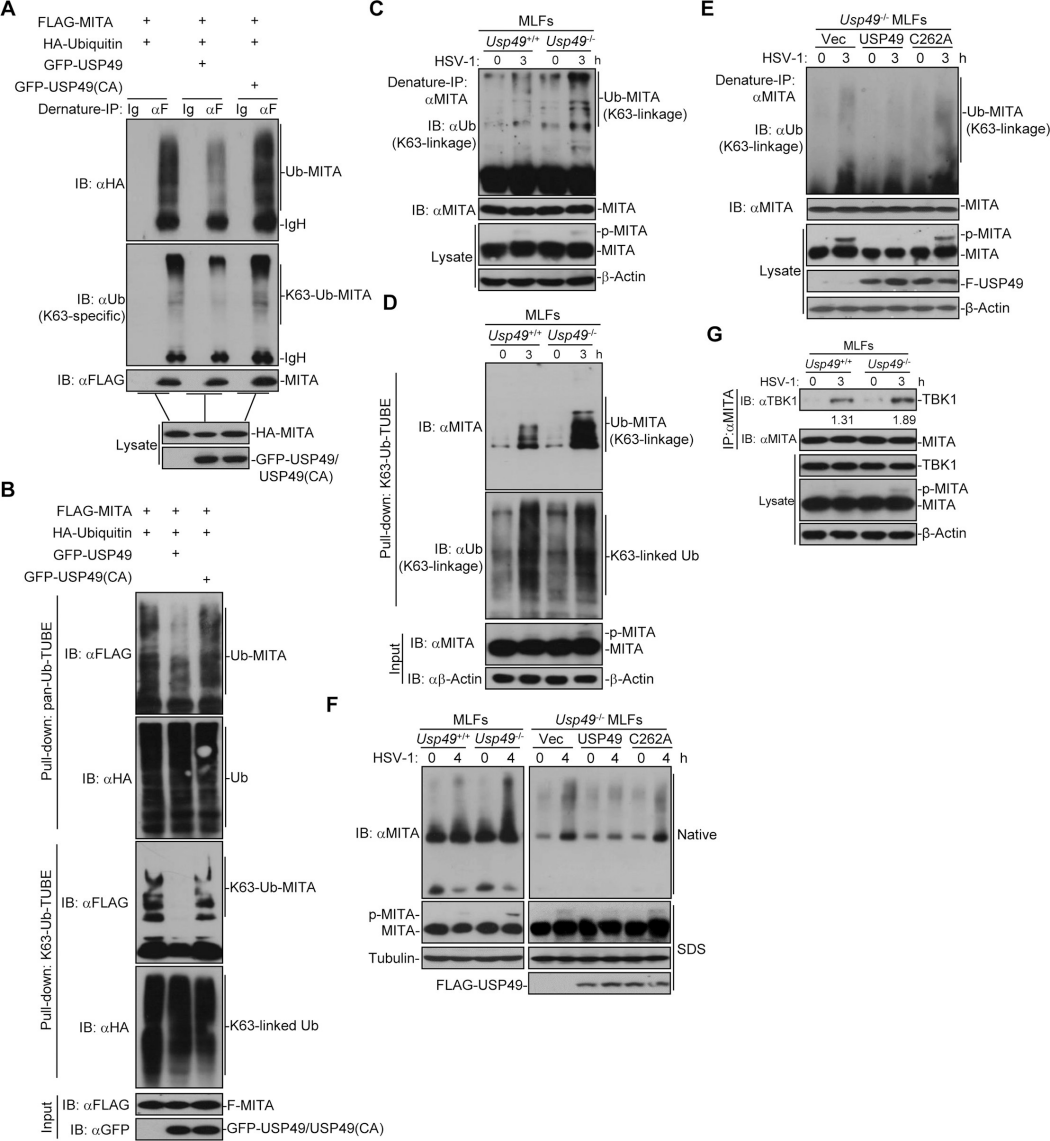
USP49 deubiquitinates and inhibits aggregation of MITA. (A) Denature-immunoprecipitation (Denature-IP) (with anti-FLAG or IgG as a control) and immunoblot analysis (with anti-FLAG, anti-HA, anti-K63-linked ubiquitin or anti-GFP) of HEK293 cells transfected with plasmids encoding FLAG-MITA, HA-Ubiquitin and empty vector or GFP-USP49 or GFP-USP49 (CA) for 24 h. (B) Pulldown (with GST beads and GST-TUBEs) and immunoblot analysis (with anti-FLAG, anti-HA or anti-GFP) of HEK293 cells transfected with plasmids encoding FLAG-MITA, HA-Ubiquitin and empty vector or GFP-USP49 or GFP-USP49 (CA) for 24 h. (C) Denature-IP (with anti-MITA) and immunoblot analysis (anti-K63-linked ubiquitin, anti-MITA or anti-β-Actin) of *Usp49*^*+/+*^ and *Usp49*^*-/-*^ MLFs infected with HSV-1 for 0–3 h. (D) Pulldown (with GST beads and TUBE) and immunoblot analysis (anti-K63-linked ubiquitin, anti-MITA or anti-β-Actin) of *Usp49*^*+/+*^ and *Usp49*^*-/-*^ MLFs infected with HSV-1 for 0–3 h. (E) Denature-IP (with anti-MITA) and immunoblot analysis (with anti-K63-linked Ub, anti-MITA, anti-FLAG or anti- anti-β-Actin) of *Usp49*^*-/-*^ MLFs reconstituted with empty vector, USP49 or USP49 (CA) infected with HSV-1 for 0–3 h. (F) Native–PAGE analysis and SDS–PAGE of the aggregation of MITA in *Usp49*^*+/+*^ and *Usp49*^*-/-*^ MLFs or *Usp49*^*-/-*^ MLFs reconstituted with empty vector, USP49 or USP49 (CA) infected with HSV-1 for 0–4 h. (G) Immunoprecipitation (with anti-MITA) and immunoblot analysis (with anti-TBK1, anti-MITA, anti-USP49 or anti-β-Actin) MLFs infected with HSV-1 for 0–4 h. Data are representative of at least three independent experiments.

Since USP49 is a MITA-interacting DUB and its deubiquitinating enzymatic activity is required for regulating antiviral signaling, we hypothesized that USP49 may eliminated polyubiquitin chains from MITA. As expected, we found that USP49 but not the enzymatic inactive mutant USP49 (C262A) catalyzed deubiquitination of MITA in cells or in vitro (*Figs 5A and S5A*). To examine the type of ubiquitin linkage on MITA that was regulated by USP49, we transfected MITA together with wild-type ubiquitin, or their mutants either retaining a single lysine residue (KO) or retaining all but one lysine residues (KR) in the presence or absence of USP49 followed by deubiquitination assays. The results showed that USP49 removed K63O- or K48R-linked but not K27O-, K48O- or K63R-linked polyubiquitin chains from MITA in cells (*S5B and S5C Fig*), indicating that USP49 primarily removes K63- but not K48-linked ubiquitination of MITA. Consistently, we observed that USP49 inhibited K63-linked ubiquitination of MITA in cells and *in vitro* by using an anti-Ub(K63-specific linkage) antibody (*Figs 5A and S5A*). We have previously demonstrated that USP18 recruits USP20 to remove K48-linked polyubiquitin chains from MITA[45]. Interestingly, we found that knockdown of USP18 did not affect USP49-mediated deubiquitinaton of MITA (*S5D Fig*), suggesting different regulatory mechanisms of K48- and K63-linked ubiquitination of MITA. We next adopted a strategy called Tandem Ubiquitin Binding Entity (TUBE) to examine the effects of USP49 on ubiquitination of MITA [44, 57–60]. As shown in Fig 5B, Pan-Ub-TUBE- and K63-Ub-TUBE-pulldown MITA were decreased by overexpression of USP49 but not USP49(C262A), suggesting that K63-linked polyubiquitin-modified MITA was deonjugated by USP49.

To further substantiate the conclusion, we next examined virus-triggered ubiquitination of MITA in the absence of USP49 and found that HSV-1 infection enhanced K63-linked ubiquitination of MITA more profoundly in *Usp49*^-/-^ MLFs than in the wild-type MLFs (*Fig 5C and 5D*). Furthermore, we found that reconstitution of USP49 but not USP49 (C262A) into *Usp49*^-/-^ MLFs inhibited HSV-1-induced K63-linked ubiquitination of MITA (*Fig 5E*). It has been reported that E3 ligases promote aggregation of signaling adaptor proteins through K63-linked ubiquitin [61]. Because USP49 removed K63-linked polyubiquitin from MITA, we examined whether USP49 affected the aggregation of MITA. As expected, USP49 deficiency in MLFs promoted the aggregation of MITA and reconstitution of USP49 but not USP49(C262A) into *Usp49*^-/-^ MLFs impaired the aggregation of MITA after HSV-1 infection (*Fig 5F*). The aggregation of MITA provides a platform to recruit kinase TBK1 which is essential for phosphorylation of MITA and subsequent recruitment of IRF3 [36]. Consistent with this notion, USP49 deficiency led to increased TBK1-MITA association and phosphorylation of MITA after HSV-1 infection (*Fig 5G*). Together these data demonstrate that USP49 deconjugates K63-linked polyubiquitin chains from MITA and inhibits MITA aggregation and the recruitment of TBK1 to the signaling platform.

## Discussion

**Fig 6:**
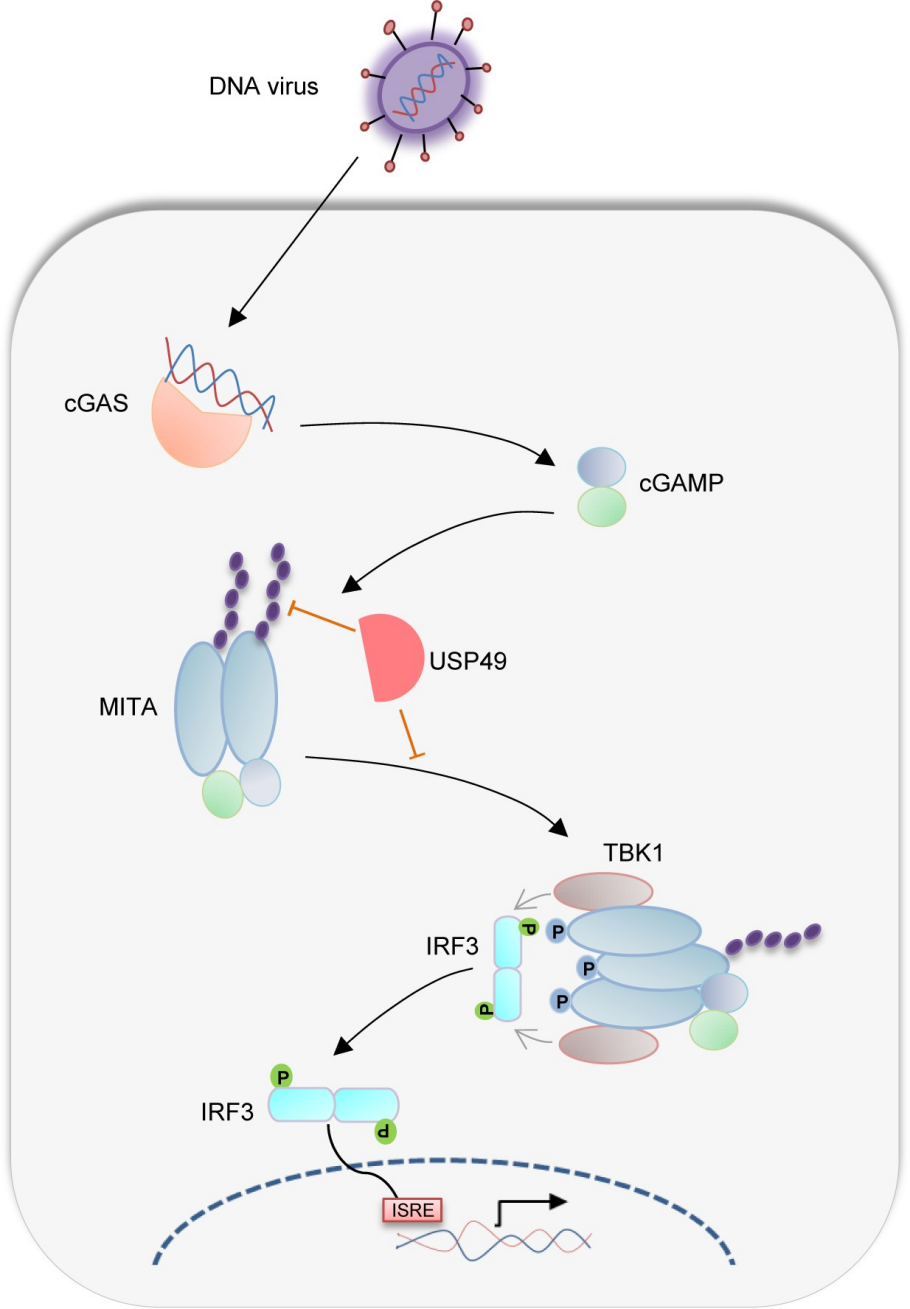
A model on USP49-mediated regulation of antiviral responses. HSV-1 infection induces production of cGAMP that binds to MITA and leads to K63-linked ubiquitination and oligomerization of MITA. USP49 removes K63-linked ubiquitin chains from MITA and thereby inhibits the oligomerization. Such a deubiquitination impairs the subsequent recruitment of TBK1 and phosphorylation of IRF3 after HSV-1 infection.

Ubiquitination and deubiquitination are reversible posttranslational modifications involved in various biological or pathological processes [62, 63]. MITA is an adaptor protein critically mediating innate immune signaling in response to cytoplasmic DNA challenge. The ubiquitination of MITA is essential for its function and stability which should be properly controlled to elicit protective immunity and avoid excessive harmful immunity [55]. AMFR promotes K27-linked ubiquitination and activation of MITA [43], whereas RNF5, TRIM30α and TRIM29 induce K48-linked ubiquitination and proteasomal degradation of MITA [39–42], which is counteracted by USP13 and USP18, USP21 or EIF3S5, respectively [23, 44–46]. Multiple E3 ligases including TRIM56, TRIM32 and MUL1 have been reported to promote K63-linked ubiquitination and aggregation of MITA [47–49]. However, the counteracting deubiquitination process is unclear. In this study, we demonstrated that USP49 interacted with MITA and deconjugated K63-linked polyubiquitin chains from MITA after HSV-1 infection, thereby downregulating MITA activation and turning down immune responses (*Fig 6*). Consistent with this notion, we found that HSV-1-induced K63-linked ubiquitination and aggregation of MITA and subsequent recruitment of TBK1 to MITA was substantially enhanced in USP49 deficient cells. USP49 deficiency also resulted in resistance to lethal HSV-1 infection, enhanced phosphorylation of TBK1, IRF3 and IκBα, increased production of type I IFNs and proinflammatory cytokines and compromised HSV-1 replication after HSV-1 infection. These findings together suggest an essential role of USP49 in the regulation of innate antiviral signaling by modulating K63-linked ubiquitination of MITA.

MITA has been implicated in defense against RNA viruses in human cell lines such as HEK293 and HeLa cells in a manner dependent on TRIM56- and/or TRIM32-mediated K63-linked ubiquitination of MITA [47, 48]. However, gene knockout studies show that neither TRIM56 nor TRIM32 deficiency in mice has any obvious effects on SeV-triggered signaling [64, 65]. Whether TRIM56 and TRIM32 function redundantly to ubiquitinate MITA and regulate RNA virus-triggered signaling remains unclear. Furthermore, infection with RNA viruses in telomerase-immortalized human foreskin fibroblasts (hTERT-BJ1) or MEFs fails to induce ubiquitination or activation of MITA [49], indicating that K63-linked ubiquitination of MITA is not required for innate immune signaling in response to RNA virus infection. Consistent with this notion, we found that knockout of USP49 had no effect on SeV-induced phosphorylation of IRF3 or IκBα and expression of downstream genes in mouse cells. In this context, it has been shown that MITA restricts RNA virus replication through inhibition of viral mRNA translation or promotion of membrane fusion-stimulated IFN production but independent of cGAMP-mediated dimerization or oligomerization [66, 67].

Although the direct genetic evidence of E3s that are responsible for K63-linked ubiquitination of MITA is still lacking, our data suggest that the K63-linked ubiquitination of MITA is essential for innate antiviral responses against HSV-1 infection. In this context, it has been reported that HSV-1 infection or cytoplasmic DNA challenge strongly induces K63-linked ubiquitination of MITA prior to its phosphorylation and translocation in both human and mouse cells [36]. Consistently, we found that knockout of USP49 potentiated HSV-1-induced K63-linked ubiquitination, aggregation and phosphorylation of MITA. Our data support and add a previously uncharacterized component in the step-wise model of MITA full activation: (i) cGAMP binding to MITA initiates dimerization of MITA; (ii) MUL1- or USP49-mediated balanced K63-linked ubiquitination of MITA modulates its aggregation and migration to ERGIC to from puncta; (iii) TBK1 is recruited to the MITA aggregate platform where TBK1 phosphorylates MITA; and (iv) such a phosphorylation leads to recruitment of IRF3 and subsequent phosphorylation by TBK1. However, it is so far unclear why USP49 interacts and counteracts K63-linked ubiquitination of MITA at early time after HSV-1 infection. A simplest explanation for this is that MITA is hyperactivated at the very early time in such a system in our study and USP49 functions as a corrector to erase the overubiquitination on MITA as a protective mechanism.

MITA-mediated signaling plays important roles in autoimmunity and tumor immunity [35, 68]. Gain-of-function mutations of MITA are found in patients with various systemic inflammatory or autoimmune diseases. A recent study on exome sequencing indicates that *USP49* is one of the frequently mutated genes associated with obesity which is recognized as a chronic inflammatory disease related to cardiovascular and respiratory diseases, type II diabetes, and cancers [69]. MITA activation by tumor DNA elicits anti-tumor immunity by type I IFN induction and tumor antigen presentation by DCs [32], whereas MITA-mediated inflammation promotes tumorigenesis in an inflammation-induced skin cancer model [70]. Interestingly, an SNP of a large linkage disequilibrium block containing *USP49* gene is associated with breast cancer survival and USP49 functions as a tumor suppressor in pancreatic cancer [53, 71]. The *Usp49*^-/-^ mice did not exhibit any autoimmune symptoms at 8 months of age which might be due to a lack of inducer of MITA activation in our housing conditions. In this context, we observed that USP49-MITA association was induced by HSV-1 infection and the basal expression of type I IFNs or proinflammatory cytokines was comparable between the wild-type and *Usp49*^-/-^ cells. Whether and how the USP49-MITA axis regulates inflammatory diseases such as colitis and tumorigenesis such as colon cancer is of great interest and requires future investigations.

## Methods

### Mice

The *Usp49*^-/-^ mice on the C57BL/6 background were generated by Nanjing Biomedical Research Institute of Nanjing University through CRISPR/Cas9-mediated gene editing. In brief, Cas9 mRNA and guide RNA (5’-AGACCACATGACTCGGAAGAGGG-3’ and 5’-AGCCACGGAAGGCGGGAATCAGG-3’) were in vitro transcribed followed by injection into the fertilized eggs that were transplanted into pseudopregnant mice. The tail DNA of F0 mice was amplified with PCR and sequenced and the chimeras were crossed with wild-type C57BL/6 mice to obtain the *Usp49*^*+*/-^ mice. The F1 *Usp49*^*+*/-^ mice were further crossed with wild-type C57BL/6 mice for at least three generations. The genotyping of the *Usp49*^*-*/-^ mice was confirmed by sequencing of the PCR fragments amplified from the genomic DNA isolated from tails using the following primers: Forward 5′-CAGAGTTGTCAGTAAGGAGT -3′ and Reverse 5′-ACCCAAGTTCACCTACACGG-3′. The age- and sex-matched *Usp49*^+/+^ and *Usp49*^*-*/-^ littermates were randomized into groups for animal studies.

### Ethics statement

All mice were housed in the specific pathogen-free animal facility at Wuhan University and all animal experiments were in accordance with protocols were adhered to the Chinese National Laboratory Animal-Guideline for Ethical Review of Animal Welfare and approved by the Institutional Animal Care and Use Committee of Wuhan University (NO. 16040B and 16060F). The mice were euthanatized with CO_2_ followed by various studies.

### Reagents and antibodies

Mouse control IgG (Santa Cruz Biotechnology, sc-2025) and rabbit control IgG (Millipore, 12–370), HRP-conjugated goat-anti mouse or rabbit IgG (Thermo Scientific, PA1-86717 and SA1-9510) (1:3000), HRP-conjugated mouse anti-FLAG (Sigma, A8592)(1:1000), mouse anti-FLAG (Sungene, KM8002)(1:2000), anti-GFP (Sungene, KM8009)(1:2000) anti-β-Actin (KM9001)(1:2000), anti-Tubulin (KM9003), anti-GAPDH(KM9002), anti-HA (COVANCE, MMS-101R)(1:2000), anti-Ubiquitin (sc-8017)(1:500), anti-ubiquitin K63-specific linkage (Millipore 05–1308)(1:500), rabbit anti-TBK1(Abcam, 96328–11), anti-p-TBK1(Abcam, 109272), anti-IRF3 (sc-9082)(1:1000), anti-p-IRF3 (Cell Singling Technologies, 4947S)(1:1000), anti-IκBα (sc-371)(1:1000), anti-p-IκBα (Cell Singling Technologies, 9246L)(1:1000), anti-USP49 (proteintech,18066-1-AP), anti-mouse MITA and anti-human MITA (Cell Singling Technologies,13647) (proteintech, 19851-1-AP) were purchased from the indicated manufactures. ISD45, DNA90, and HSV120 were previously described [44, 45, 72, 73]. ISD45: 5’-TACAGATCTACTAGTGATCTATGACTGATCTGTACATGATCTACA-3’; DNA90: 5’-TACAGATCTACTAGTGATCTATGACTGATCTGTACATGATCTACATACAGATCTACTAGTGATCTATGACTGATCTGTACATGATCTACA-3’; HSV120: 5’-AGACGGTATATTTTTGCGTTATCACTGTCCCGGATTGGACACGGTCTTGTGGGATAGGCATGCCCAGAAGGCATATTGGGTTAACCCCTTTTTATTTGTGGCGGGTTTTTTGGAGGACTT-3’.

### Generation of USP49 KO THP-1 Cells

The plasmid lentiCRISPRv2-puro (Addgene#98290) was kindly provided by Drs. Hao Yin and Ying Zhang (Wuhan University). The oligos encoding gRNA (sgRNA#1:5’-CACCGGCCTGCGGCCGCTATATTG-3’; sgRNA#2:5’-AAACCAATATAGCGGCCGCAGGCC-3’) were annealed and inserted into the lentiCRISPRv2-puro vector. The reconstituted plasmid was transfected into HEK293 cells along with the packaging vectors pSPAX2 and pMD2G. The medium was changed with fresh full medium (10% FBS, 1% streptomycin-penicillin and 10 μM β-mercaptoethanol) at 8 hours after transfection. Forty hours later, the supernatants were harvested to infect THP-1 cell lines followed by puromycin selection for two weeks for various analyses.

### Constructs

Various reporter plasmids and expression plasmids for MITA, MITA truncations, ubiquitin and ubiquitin mutants were previously described[38, 44, 45, 72, 73]. Human expression plasmids for USP49, USP49 mutants and truncations were constructed by standard molecular biology techniques. The shRNAs were synthesized and transfected with Lipofectamine 2000 according to the manufacture’s manual. Thirty-six hours after transfection, cells were harvested or stimulated followed by immunoblot or qPCR. The shRNA sequences are listed as follows:

Control, 5’- CCGGGCTGAGATGTTCCTTAGTAATCTCGAGATTACTAAGGAACATCTCAGCTTTTT-3’; shUSP49, 5’-CCGGGCCGTAATCATCGAGAGAAGACTCGAGTCTTCTCTCGATGATTACGGCTTTTT-3’;

### Quantitative RT-PCR and ELISA

Total RNA was extracted from cells or tissues using TRIzol (Invitrogen), and the first-strand cDNA was reversed-transcribed with All-in-One cDNA Synthesis SuperMix (Biotool). Gene expression was examined with a Bio-Rad CFX Connect system by a fast two-step amplification program with 2x SYBR Green Fast qPCR Master Mix (Biotool). The value obtained for each gene was normalized to that of the gene encoding β-Actin. The ELISA kits for IFN-β, IL-6 and CCL5 (BioLegend) were used to detect the indicated cytokines in the sera or cell culture supernatants.

### Co-immunoprecipitation and immunoblot analysis

The experiments were performed as previously described [44, 45, 72, 73]. In brief, cells were lysed in Nonidet P-40 lysis buffer containing 150 mM NaCl, 1 mM EDTA,1% Nonidet P-40, and 1% protease and phosphatase inhibitor cocktail (Biotool). Cell lysates were subjected to SDS-PAGE and immunoblot analysis was performed with the appropriate antibodies. For immunoprecipitation assays, the lysates were immunoprecipitated with IgG or the appropriate antibodies, and the precipitants were washed three times with lysis buffer containing 150 mM NaCl, followed by immunoblot analysis. The antibodies were diluted in 3–5% (wt/vol) fat-free milk (BD Biosciences) or 1% BSA (Sigma) in TBS (1:500–1:2,000).

### Native PAGE

The gel was pre-run with 25mm Tris and 192mm glycine, pH8.4 with 1% deoxycholate at 70 mA for 30 min. Samples in the native sample buffer (10 μg protein, 62.5 mm Tris-Cl, pH6.8, 15% glycerol and 1% DOC) were applied to the gel and electrophoresed for 30min at 70mA and 1h at 120 mA.

### Deubiquitination assays

The experiments were performed as previously described [44, 45, 72]. For deubiquitination assays in cells, cells were lysed with the lysis buffer (100 μl) and the supernatants were denatured at 95°C for 5 min in the presence of 1% SDS by lysates. The denatured lysates were diluted with lysis buffer until the concentration of SDS reduced below 0.1% followed by immunoprecipitation (denature-IP) with the indicated antibodies. The immunoprecipitants were subject to immunoblot analysis with anti-ubiquitin, or anti-K63-linked ubiquitin chains. For in vitro deubiquitination aasays, FLAG-tagged MITA and HA-tagged ubiquitin were cotransfected into HEK293 (human embryonic kidney 293, from ATCC) cells. Denature-IP was performed and the precipitants were eluted by 3 x FLAG peptide (sigma) to obtain ubiquitin-modified MITA. USP49 and USP49 (C262A) were obtained by a TNT in vitro transcription / translation kit (Promega). The ubiquitinated MITA were incubated with in vitro synthesized proteins at 37°C for 2 hours followed by overnight incubation at 16°C in the presence of 1 μM ATP. The mixture was analyzed by immunoblot with the indicated antibodies.

The plasmid encoding GST-Pan-Ub-TUBE was previously described and kindly provided by Dr. Mads Gyrd-Hansen (University of Oxford) [57]. K63-Ub-TUBE was amplified from Pet28a-Rx3(A7) (Addgene, #35525) and cloned into pGEX-T4 [58, 59]. GST-Pan-Ub-TUBE and GST-K63-Ub-TUBE were expressed in *E*. *Coli*(DE3) and purified with a GST column as previously described [44, 60, 73]. To analyze ubiquitinated MITA, GST-Pan-Ub-TUBE or GST-K63-Ub-TUBE were incubated with cell lysates and pulled down by GST beads. The GST beads were washed with lysis buffer containing 500 mM NaCl for three times and subject to PAGE electrophoresis and immunoblot analysis.

### Cell culture

The *Usp49*^+/+^ and *Usp49*^-/-^ MLFs were isolated and cultured as previously described [44, 45, 72, 73]. Bone marrow cells were isolated from femurs of *Usp49*^+/+^ and *Usp49*^-/-^ mice. The cells were cultured in DMEM containing 10% (vol/vol) FBS, 1% streptomycin-penicillin and 10 μM β-mercaptoethanol. GM-CSF and M-CSF were added to the bone marrow culture for differentiation of BMDCs and BMDMs respectively. THP-1 (human monocyte; acute monocytic leukemia), U937 (human monocytic leukemia) and HEK293 cells were from the American Type Culture Collection, authenticated by STR locus analysis and tested for mycoplasma contamination. HFF (human forehead fibroblasts) cells were kindly provided by Dr. Yan-Yi Wang from Wuhan Institute of Virology, Chinese Academy of Sciences.

### Digitonin permeabilization

The cells were treated with cGAMP (1 μg) in digitonin permeabilization solution (50 mM HEPES pH 7.0, 100 mM KCl, 3 mM MgCl2, 0.1 mM DTT, 85 mM Sucrose, 0.2% BSA, and 10 μg/ml digitonin) at 37°C for 30 minutes. The cells were then replaced with regular medium and incubated for 0–3 h followed by immunoblot or qRT-PCR analysis.

### Transfection and reporter gene assays

HEK293 cells were transiently transfected with firefly luciferase reporter (100 ng) and TK Renilla luciferase reporter (20 ng) and indicated plasmids or empty vector (100 ng) using standard calcium phosphate precipitation. After 24 h, luciferase assays were performed with a dual-specific luciferase reporter kit (Promega). The activity of firefly luciferase was normalized by that of Renilla luciferase to obtain relative luciferase activity.

### Viral infection

For qRT-PCR or immunoblot analysis, cells seeded into 24-well plates (2-5x10^5^ cells per well) or six-well plates (1x10^6^-1x10^7^ cells per well) were infected with various viruses for the indicated time points. For viral replication assays, cells (2-5x10^5^) were infected with HSV-1 or H129-G4 [56]. One hour later, the supernatants were removed and cells were washed with prewarmed PBS (1 ml) twice followed by culture in full medium for 24 hours. Viral replication was analyzed by flow cytometry, fluorescent microscopy or qRT-PCR analysis. For mice infection, age- and sex-matched *Usp49*^*+/+*^ and *Usp49*^*-/-*^ were intravenously or intranasally injected with HSV-1 (2.5 x10^6^ PFU per mouse) and the survival of animals was monitored every day. The lungs or brains were collected for qRT-PCR analysis or plaque assays at 24 hours or 4 days after infection, respectively.

### Plaque assay

The supernatants of BMDCs or MLFs cultures and the homogenates of brains from infected mice (or the serial dilutions) were used to infect monolayers of Vero cells. One hour later, the supernatants or homogenates were removed and the infected Vero cells were washed with pre-warmed PBS twice followed by incubation with DMEM containing 2% methylcellulose for 48 h. The cells were fixed with 4% paraformaldehyde for 15min and stained with 1% crystal violet for 30min before counting the plaques.

### Lentivirus -mediated gene transfer

HEK293 cells were transfected with pLKO.1-shControl, pLKO.1-shUSP49, phage-6tag-USP49, phage-6tag-USP49(C262A) or the empty vector along with the packaging vectors pSPAX2 and pMD2G. The medium was changed with fresh full medium (10% FBS, 1% streptomycin-penicillin and 10 μM β-mercaptoethanol) after 8 hours. Forty hours later, the supernatants were harvested to infect *Usp49*^*+/+*^ and *Usp49*^*-/-*^ MLFs, BMDMs or BMDCs followed by various analyses.

### Statistical analysis

Differences between experimental and control groups were tested using Student’s t-test or two-way ANOVA with Bonferroni post-test. P values less than 0.05 were considered statistically significant. For animal survival analysis, the Kaplan–Meier method was adopted to generate graphs, and the survival curves were analyzed with log-rank analysis.

## Supporting information

S1 Fig USP49 functions at the level of MITA. (A) Immunoprecipitation (with anti-MITA) and immunoblot analysis (with anti-MITA or anti-USP49) of HFF infected with HSV-1 for 0–6 hours (left panels) or U937 cells transfected with cGAMP (4 μg) for 0–2 hours (right panels).
(B) Luciferase assay analyzing ISRE promoter activity in HEK293 cells transfected with empty vector or plasmids encoding MITA, MITA plus cGAS, TBK1 or IRF3 with an empty vector or FLAG-USP49.
(C) Luciferase assay analyzing ISRE promoter activity in HEK293 cells transfected with empty vector or plasmids encoding MITA, MITA plus cGAS, TBK1 or IRF3 with an empty vector or shRNA for 24 hours (left graph). Immunoblot analysis (with anti-FLAG or anti-HA) of HEK293 cells transfected for 36 h with plasmids encoding FLAG-tagged USP49 and HA-β-Actin and either USP49-targeting shRNA or control shRNA (Con) (right panels).
Data are representative of three independent experiments (Graphs show mean ± S.D. in B and C).
(TIF)

S2 Fig Generation of *Usp49* deficient mice. (A) A scheme for CRIPSR/Cas9-mediated genome editing of the *Usp49* gene locus (left). Genotyping of Usp49 from *Usp49*^*+/+*^ and *Usp49*^*-/-*^ mice (right).
(B) Gene sequence and reading frame of *Usp49*^*+/+*^ and *Usp49*^*-/-*^ mice (left). Mice numbers of each genotype (right).
(C-E) Flow cytometry analysis of immune cells and quantitative data in thymus (C), spleen (D) and peripheral lymph nodes (E) from *Usp49*^*+/+*^ and *Usp49*^*-/-*^ mice (n = 3).
(F) Flow cytometry analysis of GM-CSF or M-CSF induced DCs or Macrophages from *Usp49*^*+/+*^ and *Usp49*^*-/-*^ mice.
Data are representative of two independent experiments (Graphs show mean ± S.D. in D-F, n = 3).
(TIF)

S3 Fig USP49 deficiency potentiates cGAMP-induced expression of downstream genes. qRT-PCR analysis of *Ifnb*, *Tnf* and *Ifna4* mRNA in *Usp49*^+/+^ and *Usp49*^-/-^ MLFs treated with digitonin-mediated cGAMP permeabilization for 0–3 h.
**P < 0.01; ***P <0.001 (analysis of two-way ANOVA followed by Bonferroni post-test). Data are representative of three independent experiments (mean ± S.D.).
(TIF)

S4 Fig Knockout of USP49 has minimal effects on SeV-triggered signaling. (A) qRT-PCR analysis of *Ifnb*, *Ifna4*, *Ccl5* and *Tnf* mRNA in *Usp49*^+/+^ and *Usp49*^-/-^ MLFs, BMDCs and BMDMs infected with HSV-1 for 0–8 h.
(B) Immunoblot analysis of phosphorylation of IRF3, IkBa, TBK1 or total IRF3, IkBa, TBK1 and β-Actin in *Usp49*^+/+^ and *Usp49*^-/-^ MLFs and BMDCs infected with HSV-1 for 0–6 hours. qRT-PCR analysis of *Usp49* mRNA in *Usp49*^+/+^ and *Usp49*^-/-^ MLFs and BMDMs infected with HSV-1 for 0–8 h.
Data are representative of three independent experiments (Graphs show mean ± S.D. in A and B).
(TIF)

S5 Fig USP49 deconjugates K63-linked ubiquitin chains from MITA. (A) In vitro deubiquitination analysis of ubiquitin-modified MITA eluted from anti-FLAG precipitates by FLAG peptide of HEK293 cells transfected with FLAG-MITA and HA-ubiquitin incubated with in vitro generated USP49 or USP49(C262A) obtained from an in vitro transcription and translation kit.
(B) Denature-IP (with anti-FLAG) and immunoblot analysis (with anti-FLAG, anti-HA or anti-GFP) of HEK293 cells transfected with plasmids encoding FLAG-MITA, HA-tagged ubiquitin mutants and either the empty vector, GFP-USP49 or GFP-USP49(CA) for 24 h.
(C) Denature-IP (with anti-FLAG) and immunoblot analysis (with anti-FLAG, anti-HA or anti-GFP) of HEK293 cells transfected with plasmids encoding FLAG-MITA, HA-tagged ubiquitin mutants and either the empty vector or GFP-USP49 for 24 h.
(D) Denature-IP (with anti-FLAG) and immunoblot analysis (with anti-FLAG, anti-HA or anti-GFP) of HEK293 cells transfected with plasmids encoding FLAG-MITA, HA-tagged ubiquitin, the empty vector or GFP-USP49 and either the control shRNA or shUSP18 for 36 h. Data are representative of three independent experiments.
(TIF)
