# Supplementary figures and images for: USP49 negatively regulates cellular antiviral responses via deconjugating K63-linked ubiquitination of MITA
Source: PLoS Pathog. 2019 Apr 3;15(4):e1007680. doi: 10.1371/journal.ppat.1007680 (PMC6464240; doi:10.1371/journal.ppat.1007680)

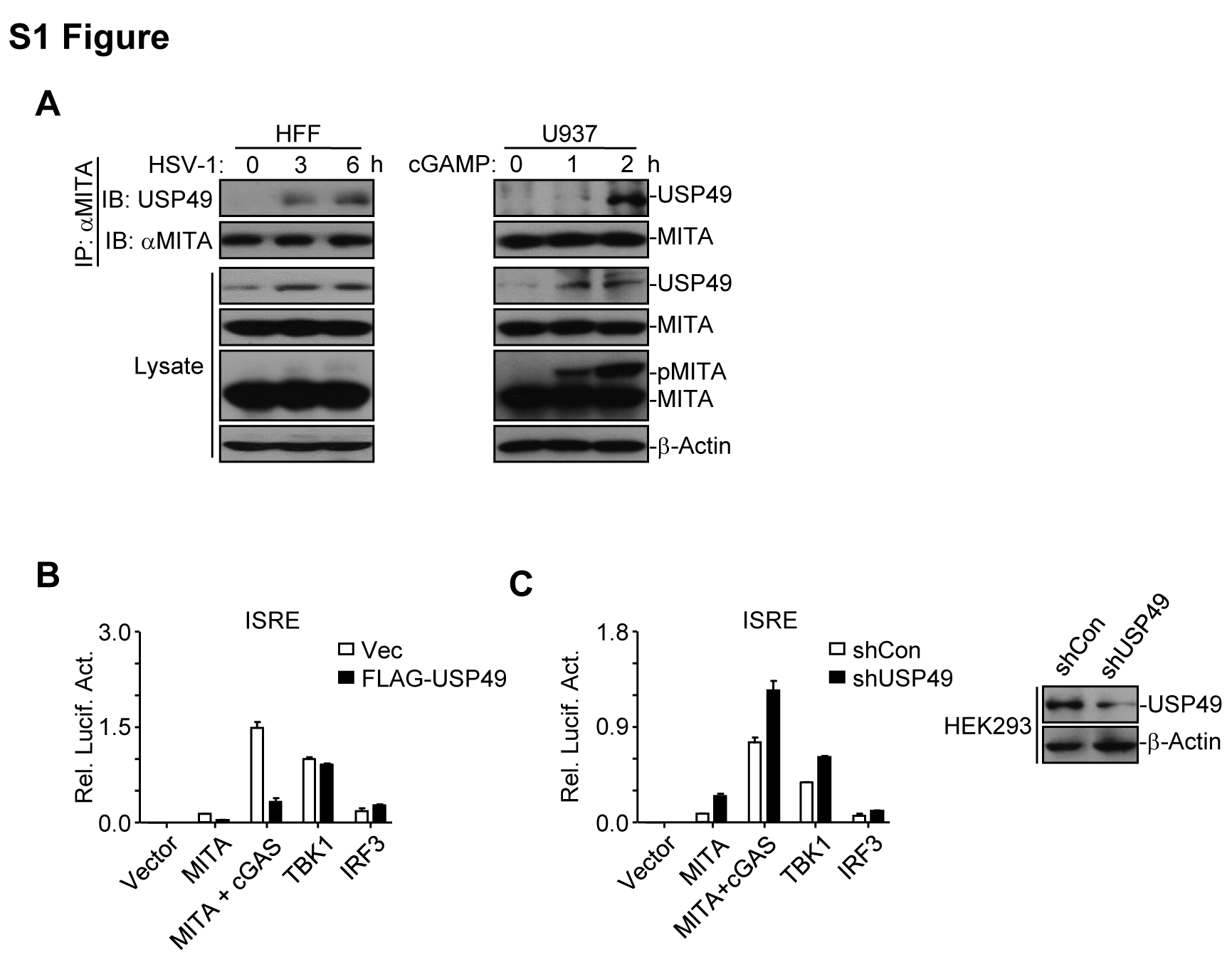

Supplement: S1 Fig — (A) Immunoprecipitation (with anti-MITA) and immunoblot analysis (with anti-MITA or anti-USP49) of HFF infected with HSV-1 for 0–6 hours (left panels) or U937 cells transfected with cGAMP (4 μg) for 0–2 hours (right panels). (B) Luciferase assay analyzing ISRE promoter activity in HEK293 cells transfected with empty vector or plasmids encoding MITA, MITA plus cGAS, TBK1 or IRF3 with an empty vector or FLAG-USP49. (C) Luciferase assay analyzing ISRE promoter activity in HEK293 cells transfected with empty vector or plasmids encoding MITA, MITA plus cGAS, TBK1 or IRF3 with an empty vector or shRNA for 24 hours (left graph). Immunoblot analysis (with anti-FLAG or anti-HA) of HEK293 cells transfected for 36 h with plasmids encoding FLAG-tagged USP49 and HA-β-Actin and either USP49-targeting shRNA or control shRNA (Con) (right panels). Data are representative of three independent experiments (Graphs show mean ± S.D. in B and C). (TIF) [file ppat.1007680.s001.tif]

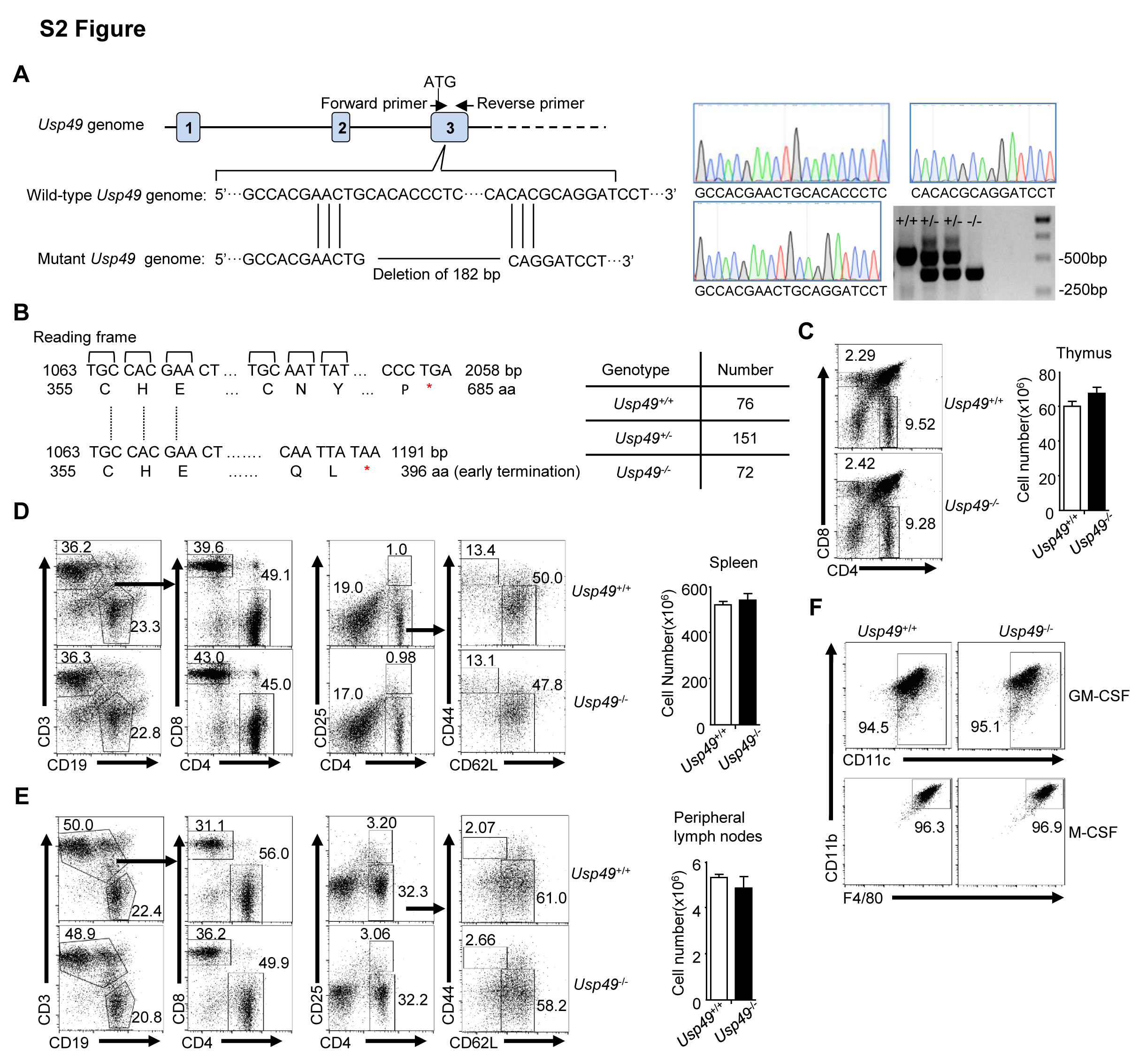

Supplement: S2 Fig — (A) A scheme for CRIPSR/Cas9-mediated genome editing of the Usp49 gene locus (left). Genotyping of Usp49 from Usp49+/+ and Usp49-/- mice (right). (B) Gene sequence and reading frame of Usp49+/+ and Usp49-/- mice (left). Mice numbers of each genotype (right). (C-E) Flow cytometry analysis of immune cells and quantitative data in thymus (C), spleen (D) and peripheral lymph nodes (E) from Usp49+/+ and Usp49-/- mice (n = 3). (F) Flow cytometry analysis of GM-CSF or M-CSF induced DCs or Macrophages from Usp49+/+ and Usp49-/- mice. Data are representative of two independent experiments (Graphs show mean ± S.D. in D-F, n = 3). (TIF) [file ppat.1007680.s002.tif]

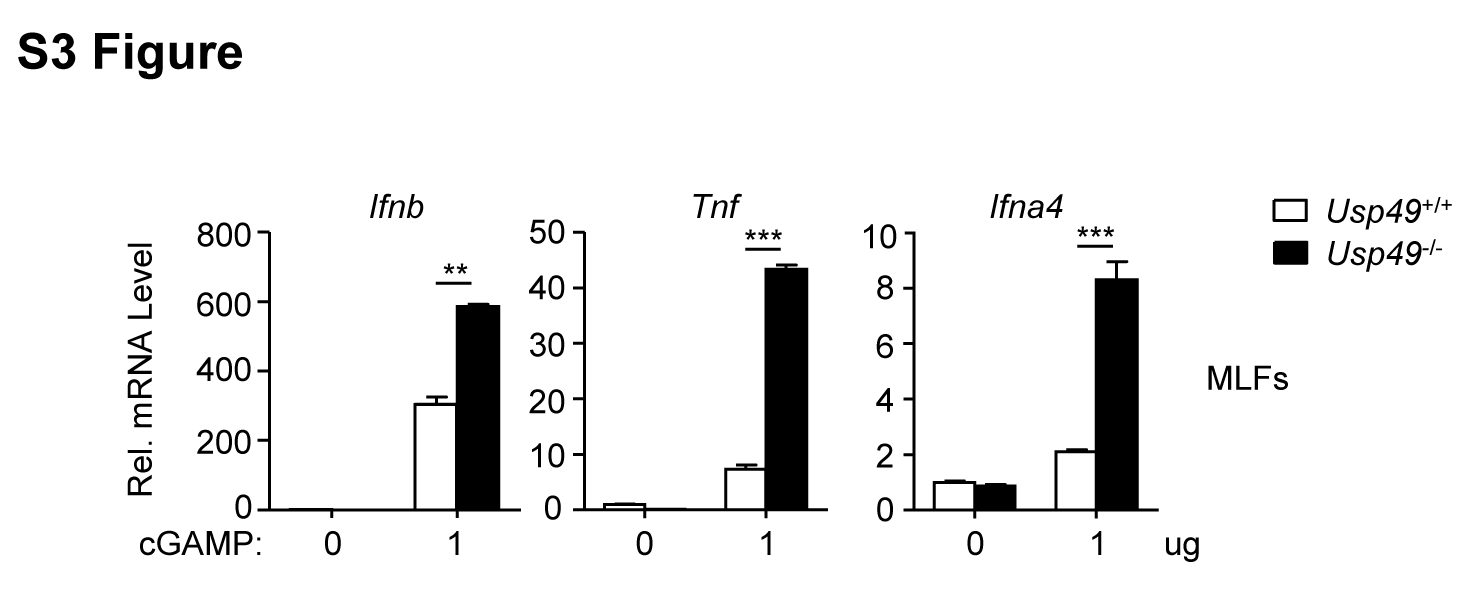

Supplement: S3 Fig — qRT-PCR analysis of Ifnb, Tnf and Ifna4 mRNA in Usp49+/+ and Usp49-/- MLFs treated with digitonin-mediated cGAMP permeabilization for 0–3 h. **P < 0.01; ***P <0.001 (analysis of two-way ANOVA followed by Bonferroni post-test). Data are representative of three independent experiments (mean ± S.D.). (TIF) [file ppat.1007680.s003.tif]

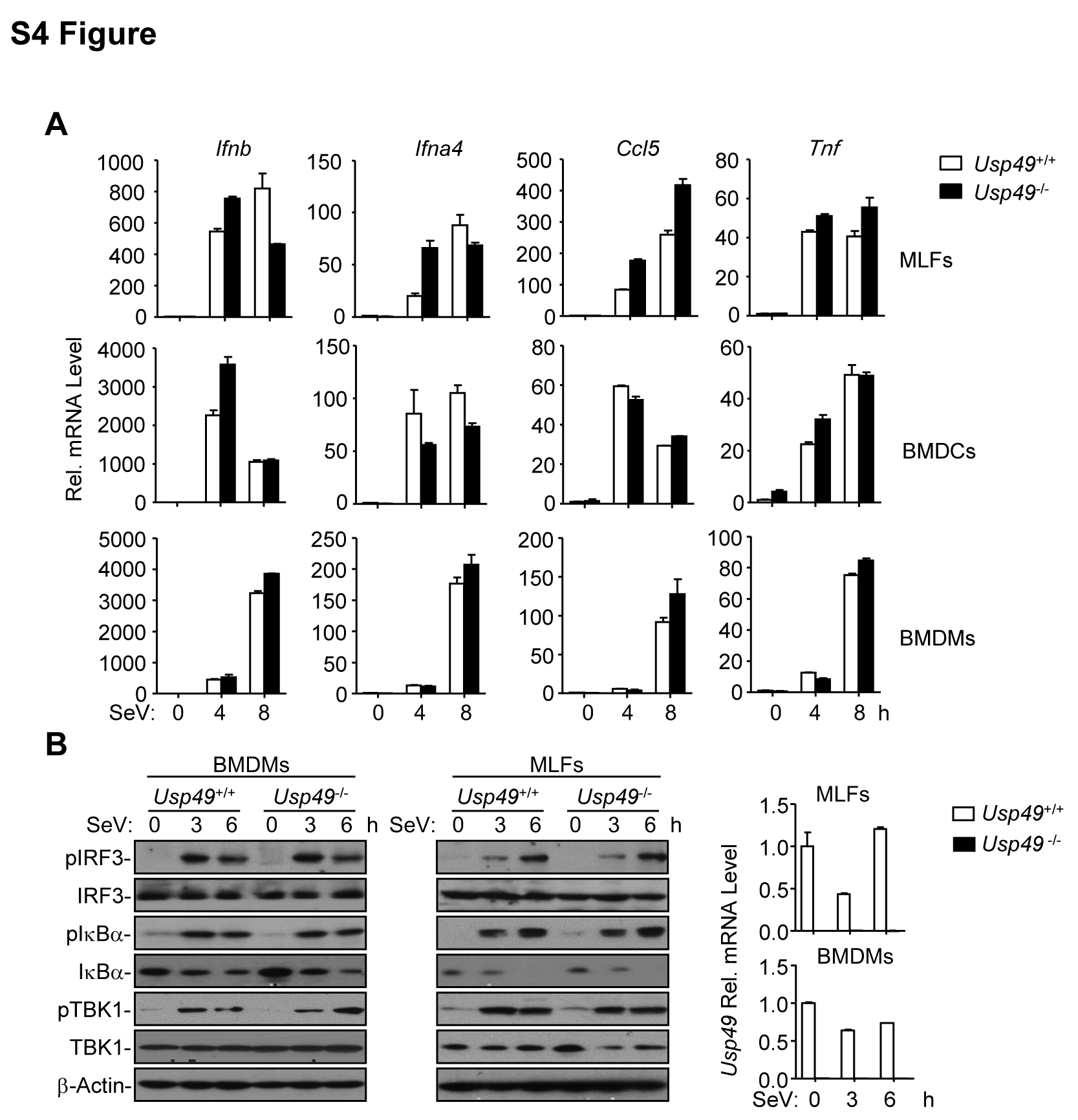

Supplement: S4 Fig — (A) qRT-PCR analysis of Ifnb, Ifna4, Ccl5 and Tnf mRNA in Usp49+/+ and Usp49-/- MLFs, BMDCs and BMDMs infected with HSV-1 for 0–8 h. (B) Immunoblot analysis of phosphorylation of IRF3, IkBa, TBK1 or total IRF3, IkBa, TBK1 and β-Actin in Usp49+/+ and Usp49-/- MLFs and BMDCs infected with HSV-1 for 0–6 hours. qRT-PCR analysis of Usp49 mRNA in Usp49+/+ and Usp49-/- MLFs and BMDMs infected with HSV-1 for 0–8 h. Data are representative of three independent experiments (Graphs show mean ± S.D. in A and B). (TIF) [file ppat.1007680.s004.tif]

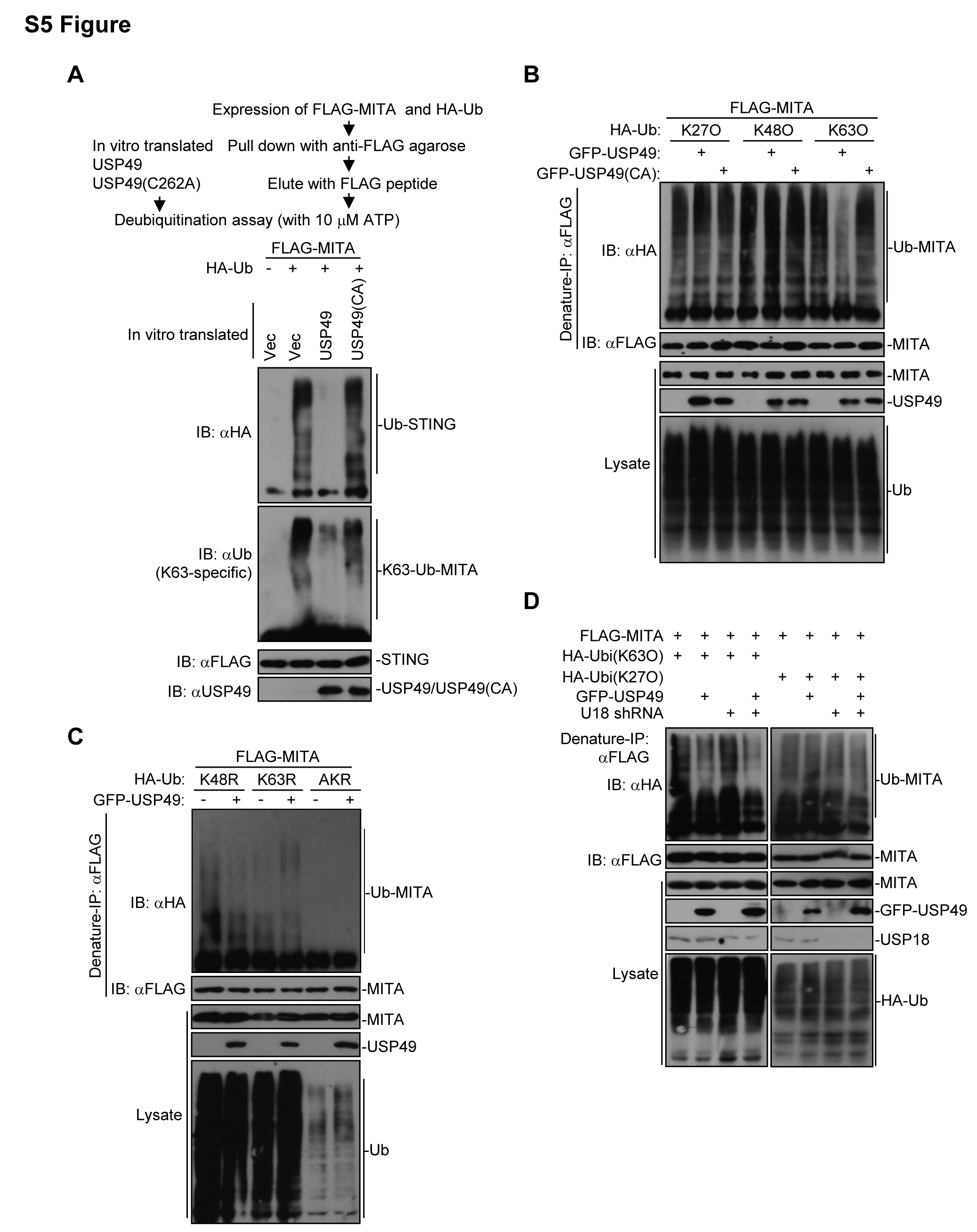

Supplement: S5 Fig — (A) In vitro deubiquitination analysis of ubiquitin-modified MITA eluted from anti-FLAG precipitates by FLAG peptide of HEK293 cells transfected with FLAG-MITA and HA-ubiquitin incubated with in vitro generated USP49 or USP49(C262A) obtained from an in vitro transcription and translation kit. (B) Denature-IP (with anti-FLAG) and immunoblot analysis (with anti-FLAG, anti-HA or anti-GFP) of HEK293 cells transfected with plasmids encoding FLAG-MITA, HA-tagged ubiquitin mutants and either the empty vector, GFP-USP49 or GFP-USP49(CA) for 24 h. (C) Denature-IP (with anti-FLAG) and immunoblot analysis (with anti-FLAG, anti-HA or anti-GFP) of HEK293 cells transfected with plasmids encoding FLAG-MITA, HA-tagged ubiquitin mutants and either the empty vector or GFP-USP49 for 24 h. (D) Denature-IP (with anti-FLAG) and immunoblot analysis (with anti-FLAG, anti-HA or anti-GFP) of HEK293 cells transfected with plasmids encoding FLAG-MITA, HA-tagged ubiquitin, the empty vector or GFP-USP49 and either the control shRNA or shUSP18 for 36 h. Data are representative of three independent experiments. (TIF) [file ppat.1007680.s005.tif]
